# Immune–Metabolic Profiling Reveals Functional Heterogeneity Within Colorectal Cancer Consensus Molecular Subtypes

**DOI:** 10.3390/biology15141128

**Published:** 2026-07-10

**Authors:** Sergio Madurga, David López-Blanco, Carles Foguet, Sara Lahoz, Helena Oliveres, Reinaldo Moreno, Teresa Gorria, Leire Pedrosa, Silvia Marin, Mariam Rojas, Jordi Camps, Francesc Mas, Joan Maurel, Marta Cascante

**Affiliations:** 1 Department of Material Science and Physical Chemistry and Research Institute of Theoretical and Computational Chemistry of University of Barcelona (IQTCUB), Universitat de Barcelona, 08028 Barcelona, Spain; s.madurga@ub.edu (S.M.); fmas@ub.edu (F.M.); 2 Centro de Investigación Biomédica en Red de Enfermedades Hepáticas y Digestivas (CIBEREHD), Instituto de Salud Carlos III (ISCIII), 28029 Madrid, Spain; dlopezbl@ub.edu (D.L.-B.); salahoz@recerca.clinic.cat (S.L.); silviamarin@ub.edu (S.M.); jcamps@clinic.cat (J.C.); 3 Department of Biochemistry and Molecular Biomedicine, Faculty of Biology, Universitat de Barcelona, 08028 Barcelona, Spain; 4 Institute of Biomedicine of Universitat de Barcelona (IBUB), Universitat de Barcelona, 08028 Barcelona, Spain; 5 British Heart Foundation Cardiovascular Epidemiology Unit, Department of Public Health and Primary Care, University of Cambridge, Cambridge CB2 0SR, UK; df545@cam.ac.uk; 6 Victor Phillip Dahdaleh Heart and Lung Research Institute, University of Cambridge, Cambridge CB2 0SR, UK; 7 Gastrointestinal and Pancreatic Oncology Group, Institut D’Investigacions Biomèdiques August Pi i Sunyer (IDIBAPS), 08036 Barcelona, Spain; 8 Translational Genomics and Targeted Therapeutics in Solid Tumors Group, IDIBAPS, Medical Oncology Department, Hospital Clínic of Barcelona, Universitat de Barcelona, 08036 Barcelona, Spain; helena.oliveres@uza.be (H.O.); rzambrano@clinic.cat (R.M.); tgorria@clinic.cat (T.G.); leire.pedrosa@iibb.csic.es (L.P.); rojasp@recerca.clinic.cat (M.R.); 9 Faculty of Medicine, Autonomous University of Barcelona, 08193 Barcelona, Spain

**Keywords:** colorectal cancer, Consensus Molecular Subtypes (CMS), IMMETCOLS, immune–metabolic profiling, tumor microenvironment, cancer metabolism, transcriptomics, tumor heterogeneity

## Abstract

The Consensus Molecular Subtype (CMS) classification provides a widely used transcriptomic framework for colorectal cancer (CRC) stratification with clear prognostic and therapeutic relevance. However, it does not fully capture the immune–metabolic heterogeneity underlying tumor–microenvironment interactions within each subtype. Here, we integrate a validated immune–metabolic gene signature as a functional layer to refine CMS classification and systematically characterize diversity across CMS1-4 tumors. Using transcriptomic data from 2918 CRC samples across three independent cohorts (GSE1, TCGA, and GSE2), we show that CMSs display robust yet distinct immune–metabolic distributions across datasets. CMS4 tumors exhibit glycolytic, stromal-dependent, and immunosuppressive profiles, whereas CMS2 and CMS3 are enriched in oxidative and metabolically flexible states. Importantly, CMS1 tumors segregate into two major immune–metabolic profiles, revealing marked heterogeneity within this immune-activated subtype. These patterns are preserved in metastatic samples, supporting their stability across disease stages. Overall, integrating immune–metabolic profiling into CMSs reveals previously unrecognized functional heterogeneity and provides a refined framework to interpret tumor–microenvironment states. This approach facilitates the identification of context-specific metabolic vulnerabilities with potential clinical relevance.

## Simple Summary

Colorectal cancer is not a single disease but includes tumors with different biological behaviors. Current molecular classifications help to group these tumors, but they do not fully explain how cancer metabolism and the immune microenvironment vary within each group. In this study, we analyzed gene expression data from 2918 colorectal cancer samples across independent patient cohorts to determine whether adding an immune–metabolic layer could improve the interpretation of established colorectal cancer subtypes. We found that immune–metabolic profiling identifies additional heterogeneity within these subtypes, including differences in metabolic activity, stromal and immune-related gene programs, chromosomal instability and patient outcome in specific groups. These patterns were also broadly observed in metastatic samples, suggesting that immune–metabolic states may remain informative across disease stages. Overall, our findings indicate that integrating immune–metabolic information with existing molecular classification systems provides a more detailed view of colorectal cancer biology. This approach may help generate hypotheses for future studies aimed at improving patient stratification and identifying context-specific therapeutic vulnerabilities, although clinical application will require further validation.

## 1. Introduction

Colorectal cancer (CRC) exhibits marked molecular and phenotypic heterogeneity, which has motivated extensive efforts to unify independent classification systems into a common transcriptomic framework [1,2]. These efforts led to the definition of four Consensus Molecular Subtypes (CMSs), which capture major biological programs and are associated with distinct clinical outcomes and tumor microenvironmental features [1,3,4]. CMS1 tumors are enriched for immune activation and microsatellite instability [1,3], CMS2 tumors display epithelial characteristics with canonical WNT and MYC signaling, CMS3 tumors show prominent metabolic alterations, and CMS4 tumors exhibit mesenchymal traits associated with TGFβ activation, angiogenesis, immune suppression and stromal infiltration [1,3,5].

Although CMS classification has proven clinically informative, important limitations remain, including residual heterogeneity within biologically related subgroups that are not fully resolved by CMSs alone [6]. In metastatic colorectal cancer, CMS stratification has nevertheless shown prognostic and predictive relevance [7,8,9]. In particular, CMSs do not fully explain the functional heterogeneity observed within tumors, especially in relation to metabolic states and tumor–microenvironment interactions. This limitation is evident in metastatic disease, where comparisons between primary tumors and metastases have revealed transcriptomic differences [10], and further analyses have suggested that CMSs reflect both tumor-intrinsic programs and microenvironmental contributions rather than fixed intrinsic identities [11,12]. In this context, CMS1 and CMS3 tumors are frequently replaced by CMS2 or CMS4 phenotypes [10,11,12].

Metabolic dysregulation is a central feature of colorectal cancer biology and has been linked to tumor initiation, growth, molecular classification and clinical behavior [13]. Although metabolic alterations have been most extensively characterized in CMS3 tumors, the metabolic features of the remaining CMSs, as well as their relationship with immune and stromal components of the tumor microenvironment, remain incompletely defined. Increasing evidence indicates that metabolic programs and immune contexture are tightly interconnected and jointly shape tumor progression and tumor–microenvironment dependencies [14].

In recent years, several approaches have sought to refine the CMS framework by incorporating features of the tumor microenvironment, including immune and stromal components. These include classifications based on immune infiltration and stromal enrichment, as well as studies integrating tumor-intrinsic and microenvironmental signals within CMSs [3,11,12]. These frameworks have provided important insights into the contribution of non-tumor compartments to CRC heterogeneity, highlighting the role of immune activity and stromal interactions in shaping tumor phenotypes. However, these approaches largely focus on compositional aspects of the tumor microenvironment and do not explicitly account for the functional interplay between metabolic programs and immune-related processes. Given the growing recognition that metabolic reprogramming and immune contexture are tightly interconnected [14], there remains a need for frameworks that integrate these dimensions into a unified transcriptional perspective. In this context, incorporating immune–metabolic features may provide complementary information to existing CMS-based stratifications by capturing coordinated functional states that are not resolved by immune- or stroma-centered classifications alone.

In this study, we investigated whether adding IMMETCOLS [15], a previously established transcriptomic classifier based on genes linked to immune and metabolic processes, could complement the functional interpretation of the CMS framework. Briefly, the three IMMETCOLS clusters are defined as follows: IMC1 represents an immune- and stromal-enriched immune–metabolic state characterized by increased expression of glycolytic, hypoxia-related, extracellular matrix, TGFβ and immune checkpoint-associated transcriptional programs. IMC2 is characterized by transcriptional features associated with glutamine metabolism, lipid/peroxisomal pathways, autophagy and lysosomal programs, consistent with an adaptive metabolic transcriptional state. IMC3 displays higher expression of oxidative phosphorylation, mitochondrial metabolism, one-carbon metabolism and proliferative/antioxidant-related transcriptional programs. In the present study, these IMMETCOLS clusters were integrated with CMS classification to explore immune–metabolic heterogeneity within colorectal cancer subtypes. We applied this signature to characterize heterogeneity within CMS1-4 tumors and to assess whether its distributions were conserved across primary and metastatic samples. By analyzing 2918 CRC samples from three independent public cohorts, including primary and metastatic tumors, we assessed how immune–metabolic profiles distribute across CMSs and whether these patterns are conserved across disease stages.

## 2. Materials and Methods

### 2.1. CRC Dataset Collection

#### 2.1.1. GSE1 Set

For the GSE1 set, we analyzed and integrated eight cohorts with gene expression microarray and clinical and phenotypic data on 1328 CRC samples. These cohorts are accessible from the Gene Expression Omnibus (GEO) repository under the accession numbers GSE14333, GSE17536, GSE31595, GSE33113, GSE38832, GSE39084, GSE39582, and GSE17537. For all series, raw gene expression profiles were obtained from the Affymetrix GeneChip U133 Plus 2.0 for *Homo sapiens*. All samples selected had information on survival. In addition, other clinical and phenotypic information was evaluated, such as age, sex, tumor location, TNM staging, presence of *KRAS* and *BRAF* mutations, and microsatellite status, if available. The probe sets of the arrays were obtained with the GEOquery R package (v2.70.0). They were then mapped to the expression signal of 20,852 human genes and normalized using the Bioconductor/R packages affy (v1.80.0), gcrma (v2.74.0), and hgu133plus2.db (v3.13.0). Potential batch effects were removed using the ComBat method implemented in the sva R package (v.3.50.0). As in Guiney et al. [1], when different probe sets corresponded to the same gene, this gene was represented by the probe set with the largest mean absolute deviation.

#### 2.1.2. TCGA Set

TCGA RNAseq data, alongside clinical, survival, and mutation data for CRC samples from the TCGA, were obtained from UCSC Xena [16]. In total, data on 456 primary tumor samples were used. Gene counts were normalized using the variance-stabilizing transformation (VST) function of the DESeq2 package for R. Samples were considered KRAS- or BRAF-mutant if they had a missense mutation in *KRAS* or *BRAF*, respectively, reported in the VarScan2 Variant Aggregation and Masking output available in UCSC Xena.

Copy number alteration (CNA) segments from SNP6.0 array data were downloaded from the Genome Data Commons (GDC) portal (https://portal.gdc.cancer.gov/, accessed on 15 February 2023) for the TCGA COAD cohort (*n* = 456 primary tumors). For each tumor, the CNA burden was calculated as the fraction of the genome containing copy number segments with log2 ratios > |0.2| divided by the total length of all segments of the genome.

#### 2.1.3. GSE2 Set

We also evaluated a second validation cohort with gene expression microarray data and clinical and phenotypic information from 1134 CRC samples from GSE131418 (hereafter referred to as GSE2). This cohort comprises two subcohorts, here referred to as MCC and Consortium, and includes both primary (PRIM) and metastatic (MET) tumor samples (*n* = 878 primary samples, with *n* = 545 from MCC and *n* = 333 from Consortium, and *n* = 257 metastatic samples, with *n* = 184 from MCC and *n* = 73 from Consortium). This distinction allowed for stratification of results by tumor origin in GSE2, which was not possible in GSE1 and TCGA datasets as they include only primary tumors. Sample GSM3777007 was excluded due to the lack of phenotypic information.

Gene expression was determined using HuRSTA-2a520709 Affymetrix arrays (GPL15048). MCC and Consortium subcohorts were processed separately. Each group was normalized using the Robust Multi-array Average (RMA) method implemented in the affy R package (v1.80.0), and the ComBat method, implemented in the sva R package (v3.50.0), was applied to correct for potential batch effects between primary and metastatic samples.

### 2.2. CMS Classification

For each dataset, colorectal cancer samples were first classified into Consensus Molecular Subtypes (CMSs) using the CMS Classifier v1.0.0 R package and the single sample predictor (SSP) classifier [1]. Subsequently, normalized expression values of the genes included in the previously described IMMETCOLS immune–metabolic signature (*ENTPD1*, *FAP*, *GLS*, *GLUL*, *GOT1*, *LDHA*, *TGFB1*, *TWIST1*, *ZEB1* and *ZEB2*) were used to assign samples to immune–metabolic clusters through a neural network classifier [15].

### 2.3. Survival Analysis

Overall survival (OS) was analyzed according to the Kaplan–Meier method, implemented with the KaplanMeierFitter function from the lifelines (v0.30.0) Python package. We excluded 79 samples from the GSE1 cohort that either lacked survival data or did not exhibit comparable data distributions after normalization. The presence of batch effects was also investigated using principal component analysis (PCA). The PCA results confirmed that all series were equally distributed. The log-rank test was used to assess significant differences between pairs of survival distributions with the log ranktest Python function of the lifelines package. Hazard rates were obtained from the Cox hazard regression models. The Cox model was fitted using the CoxPHFitter function from the Python lifelines package for calculation of multivariate models.

### 2.4. Differential Gene Expression

Overall volcano plots were generated to analyze the effect of subdividing the IMC1 group based on *ACTA2* and *IL1A* gene expression in the GSE1 cohort. High- and low-expression groups for *ACTA2* and *IL1A* were defined by selecting the top 25% and bottom 25% of cases for each gene, respectively.

For gene-level analyses, differential expression between groups was assessed using Welch’s *t*-test implemented in the scipy.stats Python module (SciPy v1.16.3). The difference in expression was calculated as the difference in mean log2-transformed expression values between high and low groups (Δlog2 expression). *p*-values were adjusted for multiple testing using the Benjamini–Hochberg procedure, implemented with the statsmodels Python package. Features with a false discovery rate (FDR-BH) < 0.05 were considered statistically significant. For gene-level plots, an additional threshold on absolute log2 expression differences (|Δlog2 expression|) was applied as indicated. Volcano plots were generated using log2 expression differences on the x-axis and −log10(FDR-BH) on the y-axis. Significant features were highlighted in red.

### 2.5. Gene Sets

For the visualization of metabolic features beyond the IMMETCOLS signature, a broader panel of metabolism-related genes was analyzed. This panel was defined by combining pathway-based selection of genes representative of key metabolic processes (including glycolysis, oxidative phosphorylation, amino acid metabolism and lipid metabolism) with data-driven identification of genes differentially expressed across IMMETCOLS clusters. Selected genes were further filtered based on their functional relevance in cancer metabolism.

## 3. Results

### 3.1. CMS and IMMETCOLS Distributions in Primary and Metastatic Samples

**Figure 1:**
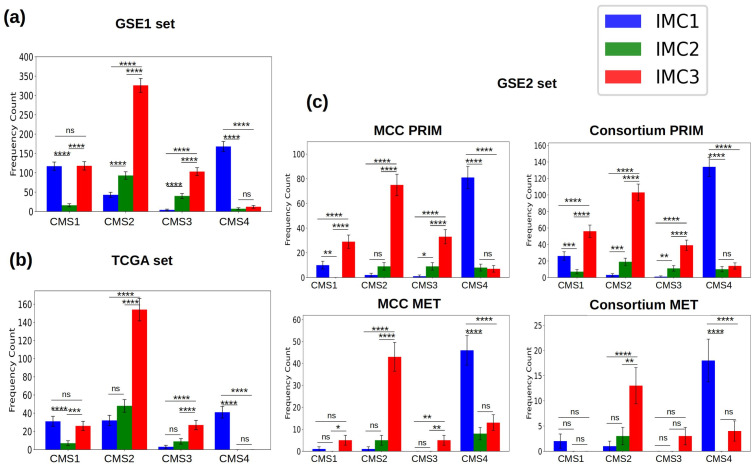
Distribution of IMC clusters and CMS classification. (**a**) Distribution for patients of the GSE1 sets (*n* = 1328). (**b**) Distribution for patients of the TCGA set (*n* = 456). (**c**) Distribution for patients of the GSE2 set (*n* = 1135). Fisher’s Exact Test was employed to evaluate the statistical significance of differences between the frequency counts of IMC categories within each CMS. Significance is indicated as follows: * *p* < 0.05; ** *p* < 0.01; *** *p* < 0.001; **** *p* < 0.0001; ns, not statistically significant. The samples not classified by the CMS classifier are not displayed in the figures and correspond to 281, 78 and 276 samples for the GSE1, TCGA and GSE2 sets, respectively. PRIM: primary; MET: metastatic.

The distribution of IMMETCOLS profiles across Consensus Molecular Subtypes (CMSs) was highly consistent across the GSE1, TCGA and GSE2 cohorts (Figure 1 and Tables S1 and S2). While each CMS showed a preferential association with specific IMMETCOLS clusters, this classification revealed substantial intra-subtype heterogeneity.

CMS4 tumors were predominantly associated with the IMC1 immune–metabolic profile, whereas CMS2 and CMS3 tumors were mainly enriched in IMC3 and, to a lesser extent, IMC2 profiles. Notably, CMS1 tumors segregated into two major immune–metabolic states, with a comparable representation of IMC1 and IMC3, indicating substantial immune–metabolic heterogeneity within a transcriptomically immune-activated CMS.

Importantly, the same immune–metabolic distributions within CMSs were preserved in metastatic samples from the MCC and Consortium cohorts (*n* = 257) (Figure 1 and Table S2), supporting the robustness of these associations across disease stages.

### 3.2. Overall Survival in Limited Disease

**Figure 2:**
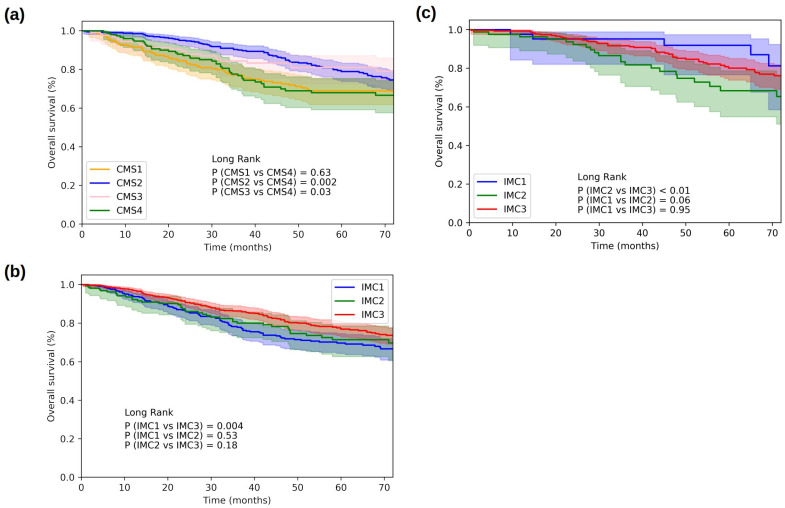
Kaplan–Meier plots of the survival analysis. (**a**) Kaplan–Meier plots of the survival analysis of the GSE1 set for CRC patients subdivided into CMS1, CMS2, CMS3, and CMS4 groups; *n* = 232, 415, 128 and 153 for CMS1, CMS2, CMS3 and CMS4, respectively. (**b**) Kaplan–Meier plots of the survival analysis of the GSE1 set subdivided into IMC1, IMC2, and IMC3 groups; *n* = 377, 160 and 626 for IMC1, IMC2 and IMC3, respectively. (**c**) Kaplan–Meier plots of the survival analysis of the CMS2 group of the GSE1 set subdivided into IMC1, IMC2 and IMC3 groups; *n* = 42, 85 and 288 for IMC1, IMC2 and IMC3, respectively. Shaded areas represent 95% confidence intervals.

To assess whether the immune–metabolic heterogeneity identified within CMSs has prognostic relevance, overall survival (OS) was evaluated in the GSE1 cohort using Kaplan–Meier analysis and log-rank testing (Figure 2).

Consistent with previous reports, CMS classification identified significant survival differences, with CMS4 patients showing worse OS compared with CMS3 (*p* = 0.03) and CMS2 (*p* = 0.002). Analysis based on IMMETCOLS stratification further revealed that IMC1 was associated with significantly worse OS compared with IMC3 (*p* = 0.004).

We next examined whether immune–metabolic classification could refine survival prediction within individual CMSs. This analysis was restricted to patients with limited disease (stages I–III), as the prognostic value of CMSs has been established primarily in this setting and the number of stage IV cases was insufficient for reliable evaluation.

Within CMS2 stage I–III tumors, patients classified as IMC2 exhibited worse overall survival compared with those classified as IMC3. Subgroup sizes were IMC1 (*n* = 42), IMC2 (*n* = 85), and IMC3 (*n* = 288). Pairwise log-rank tests showed *p* = 0.056 for IMC1 vs. IMC2, *p* = 0.949 for IMC1 vs. IMC3, and *p* = 0.0097 for IMC2 vs. IMC3. In an unadjusted Cox model using IMC2 as the reference, IMC3 was associated with improved overall survival (HR = 0.53, 95% CI = 0.33–0.85, *p* = 0.009). The proportional hazards assumption was assessed using Schoenfeld residuals, with no evidence of violation. This association was retained in the multivariate model adjusted for age, stage and tumor sidedness (HR = 0.50, 95% CI = 0.30–0.83, *p* = 0.01; Table S3). Although IMMETCOLS stratification identified prognostic differences within CMS2 tumors, the subgroup sizes were limited, particularly for CMS2-IMC1 and CMS2-IMC2. Therefore, these survival findings should be considered exploratory and require validation in larger independent cohorts.

### 3.3. Metabolic Features Associated with IMMETCOLS Clusters in Primary and Metastatic Samples

**Figure 3:**
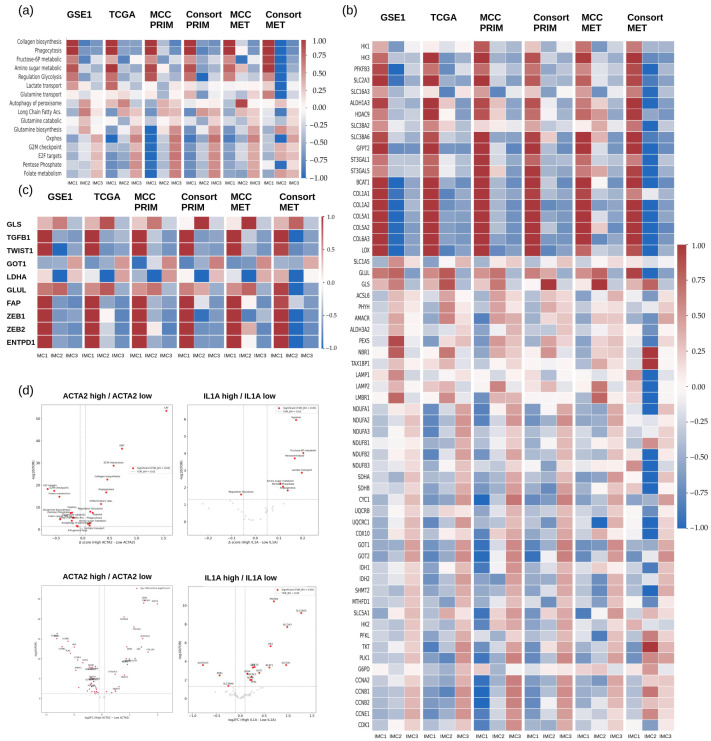
Metabolism-related characteristics according to IMC1, IMC2, and IMC3 groups. (**a**) Gene expression of selected pathways for the GSE1, TCGA, and GSE2 sets classified into IMMETCOLS clusters. (**b**) Heatmap of patients of the GSE1, TCGA, and GSE2 sets classified into IMMETCOLS clusters according to the expression of key enzymes. (**c**) Heatmap depicting the average gene expression of the 10 IMC genes among the three IMC groups for the GSE1 and TCGA sets. Gene expression values are range-scaled between ±1. GSE1, *n* = 1328 samples; TCGA, *n* = 456 samples; and GSE2, *n* = 1134 samples. (**d**) Differential pathway score and gene expression plots showing the effect of subdividing the IMC1 group according to *ACTA2* and *IL1A* expression. Red points indicate significant pathways or genes after Benjamini–Hochberg correction (FDR-BH < 0.05); an additional |log2FC| threshold was applied in gene-level plots where indicated. PRIM: primary; MET: metastatic.

To provide a functional interpretation of the heterogeneity observed across CMSs, we next examined the metabolic programs associated with each of the three clusters defined by IMMETCOLS. Metabolic pathways and key genes overexpressed in each cluster are shown in Figure 3a–c and Figure S1A,B. A broader panel of metabolism-related genes, selected based on differential expression across IMMETCOLS clusters and their biological relevance, was used to further characterize metabolic features (Figure 3b). While the IMMETCOLS classification was originally defined across multiple tumor types, here we focus specifically on colorectal cancer, enabling a more detailed characterization of the metabolic programs associated with each cluster in this tumor type.

IMC1 tumors displayed high expression of genes (Figure 3b and Figure S2) involved in glycolysis and in the transport of glucose, lactate and branched-chain keto acids (BCKAs). This metabolic configuration is associated with transcriptional signatures linked to stromal and immune-related processes [17]. Amino sugar metabolic pathways and glutamine transporters were also upregulated, conferring an increased capacity for hexosamine and sialic acid synthesis required for protein glycosylation [18]. Notably, this metabolic pattern was consistently observed in both primary and metastatic samples (Table S4).

Further stratification of IMC1 tumors based on *IL1A* and *ACTA2* expression revealed additional inflammatory and stromal heterogeneity. *IL1A*-high and *ACTA2*-high IMC1 tumors displayed transcriptional features reminiscent of previously described CAF-related programs [19]; however, their correspondence to specific CAF subtypes remains exploratory and requires validation using single-cell, spatial, or protein-level approaches. This interpretation is consistent with observations reported in other tumor types, including pancreatic cancer, and is in line with metabolic features previously described for IMMETCOLS clusters in pan-cancer studies. IMC1 *IL1A*-high samples were enriched for hypoxia-related pathways and lactate transport, whereas IMC1 *ACTA2*-high samples showed enrichment of epithelial–mesenchymal transition (EMT) and collagen biosynthesis programs (Figure 3d). At the transcriptomic level, *PFKFB3* and the lactate transporter MCT4 (*SLC16A3*) were strongly overexpressed in *IL1A*-high samples, while *ZEB1*, a regulator of collagen synthesis and deposition [20], and *GFPT2*, a key enzyme in fibroblast hexosamine metabolism [21], were preferentially upregulated in *ACTA2*-high tumors (Table S4). Within IMC1 tumors, the distinction between *IL1A*-high and *ACTA2*-high cases suggests the presence of additional stromal and inflammatory heterogeneity. However, this subdivision should be considered exploratory. Although these groups display transcriptional features reminiscent of previously described CAF-related programs, bulk RNA-seq data do not allow us to directly assign these signals to specific fibroblast populations. Additional validation using single-cell transcriptomic datasets, published CAF signatures, spatial profiling, or protein-level assessment of CAF markers will be required to determine whether these IMC1 subgroups correspond to distinct functional CAF states. In addition, the immune–metabolic patterns identified in our study should be interpreted in the context of growing evidence that metabolic reprogramming of the CRC tumor immune microenvironment can influence immune escape and response to immunotherapy. This is particularly relevant given that combinations of immune checkpoint inhibitors with strategies targeting aerobic glycolysis or other metabolic pathways are being actively investigated [22]. Nevertheless, the therapeutic relevance of IMC-associated states remains exploratory and requires validation in therapeutically annotated cohorts and functional experimental models.

IMC2 tumors were characterized by reduced OXPHOS-related gene expression and increased expression of *GLUL*, together with enrichment of genes involved in peroxisomal lipid metabolism, autophagy and lysosomal processes. These transcriptional features are compatible with adaptive metabolic programs that may support survival in metabolically constrained tumor microenvironments, although functional validation would be required to confirm this interpretation. Genes involved in long-chain fatty acid metabolism, peroxisomal α/β-oxidation and peroxisomal protein import were also upregulated, suggesting alternative utilization of glutamine and lipid substrates. Autophagy-related genes and lysosomal structural proteins were concomitantly overexpressed. Although metastatic IMC2 tumors retained these transcriptional features, the relative frequency of this subtype was markedly reduced in metastatic samples, representing approximately 5% and 11% of cases in the MCC and Consortium metastatic samples of the GSE2 dataset (Figure 1; Table S2).

IMC3 tumors exhibited increased expression of genes encoding components of mitochondrial respiratory chain complexes I-IV compared with the other IMMETCOLS clusters, indicating enrichment of OXPHOS-related transcriptional programs. Upregulation of genes involved in folate-mediated one-carbon metabolism, glutamine transport, aspartate aminotransferases and isocitrate dehydrogenases suggests enrichment of mitochondrial and biosynthetic metabolic gene programs [23,24]. However, these transcriptomic patterns do not directly demonstrate metabolic flux, substrate utilization or reductive carboxylation activity. Genes from the upper glycolytic pathway and the pentose phosphate pathway (PPP) were also enriched, supporting the generation of biosynthetic precursors for proliferation. Polo-like kinase 1 (*PLK1*), a direct activator of PPP flux [25], was overexpressed, together with genes associated with the G2/M checkpoint and E2F targets, consistent with an actively cycling phenotype. This metabolic program was conserved across primary and metastatic samples (Table S4). Because metabolic programs in this study were inferred from bulk transcriptomic data, they should be interpreted as pathway-level gene expression patterns rather than direct evidence of metabolic flux or substrate utilization. Functional validation using metabolic flux analysis, isotope tracing or metabolomics would be required to confirm whether IMC3 tumors exhibit increased metabolic flexibility or reductive carboxylation activity.

### 3.4. Immune Microenvironment-Related Features and Chromosomal Instability Across IMMETCOLS Clusters

To further characterize the biological features associated with IMMETCOLS clusters, we examined transcriptomic signatures related to the tumor microenvironment and chromosomal instability. Tumors were also stratified according to microsatellite instability status, including microsatellite-stable (MSS) and microsatellite instability-high (MSI-H) cases, which reflect mismatch repair proficiency and deficiency, respectively.

**Figure 4:**
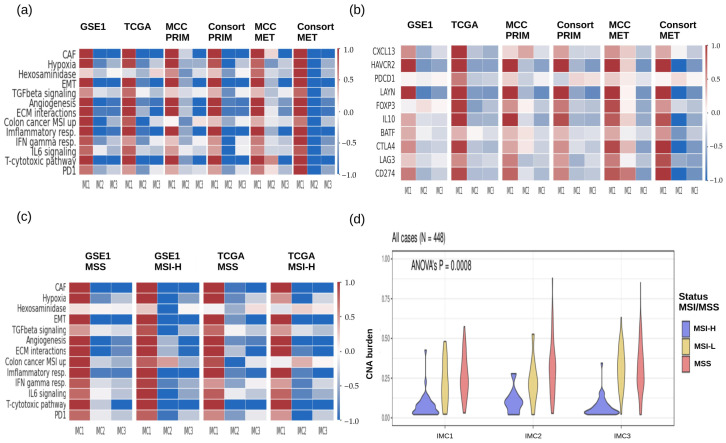
Immune microenvironments and chromosome instability characteristics. (**a**) Gene expression of selected pathways for the GSE1, TCGA, and GSE2 sets classified into IMMETCOLS clusters; *n* = 1328, 456 and 1134 for GSE1, TCGA and GSE2, respectively. Colors indicate the relative signature expression (z-score). (**b**) Heatmap of samples of the GSE1, TCGA, and GSE2 sets classified into IMMETCOLS clusters according to the expression of key enzymes. (**c**) Selected pathway distribution according to microsatellite instability status (MSS and MSI-H) in the GSE1 and TCGA sets. (**d**) Distribution of CNA burden stratified by microsatellite instability status including MSI-L (microsatellite instability-low), an intermediate category between MSS and MSI-H; *n* = 489 and *n* = 86 GSE1 samples for MSS and MSI-H, respectively; *n* = 364 and 83 TCGA samples for MSS and MSI-H, respectively.

IMC1 tumors showed increased expression of gene programs associated with extracellular matrix organization, EMT, TGFβ signaling, hypoxia and angiogenesis (Figure 4a). In addition, these tumors displayed elevated expression of genes commonly associated with immune-related pathways. These observations are consistent with a microenvironment characterized by strong stromal and immune-related transcriptional signals. These features are consistent with those previously described for IMMETCOLS clusters in the original study, and are used here to facilitate the interpretation of transcriptomic programs associated with each cluster in colorectal cancer.

Among immune-related features, IMC1 tumors exhibited increased expression of markers commonly linked to T cell exhaustion, including PD1, *ENTPD1* (CD39), *HAVCR2* (TIM3) and *LAG3* (Figure 4b). While these markers are frequently associated with exhausted T cell states [26,27,28,29], their expression here reflects transcriptomic enrichment rather than direct measurement of specific immune cell populations. Although IMC1 tumors displayed increased bulk RNA expression of genes commonly associated with inhibitory immune checkpoints and T cell dysfunction/exhaustion-related programs, these data should be interpreted with caution. Bulk transcriptomic analysis does not allow for attribution of these markers to specific immune cell subsets, and expression of PD1, *LAG3* or *HAVCR2*/TIM3 does not by itself demonstrate functional T cell exhaustion. Additional single-cell, spatial or functional immune profiling would be required to confirm the presence and functional relevance of exhausted T cell populations in these tumors.

In contrast, IMC2 and IMC3 tumors generally showed lower expression of these immune- and stroma-related signatures, suggesting reduced prominence of these transcriptional programs compared with IMC1. These patterns were also observed within microsatellite instability-high (MSI-H) tumors, indicating that differences identified by IMMETCOLS are not solely explained by mismatch repair status (Figure 4c; Table S4).

To explore potential associations with genomic instability, we quantified copy number alteration (CNA) burden using SNP array data. The three IMMETCOLS clusters exhibited significant differences in CNA burden (ANOVA *p* = 0.0008), with median values of 19.3% for IMC1, 28% for IMC2 and 26.1% for IMC3 (Figure 4d). CNA burden differed significantly across IMMETCOLS clusters, with IMC2 and IMC3 tumors showing higher median CNA burden than IMC1 tumors. In the TCGA cohort, where CNA data were available, CMS2 tumors were predominantly assigned to IMC3 and IMC2, whereas CMS4 tumors were mainly assigned to IMC1. Thus, CNA burden varied according to IMC state and paralleled the differential distribution of IMC states across CMS2 and CMS4 tumors.

## 4. Discussion

This study demonstrates that integrating immune–metabolic information into the established Consensus Molecular Subtype (CMS) framework provides additional functional insight into colorectal cancer heterogeneity. Rather than proposing a new classification, immune–metabolic profiling reveals biologically meaningful differences in metabolic programs, tumor-microenvironment-related transcriptional features and clinical behavior within CMSs. Importantly, these immune–metabolic patterns were conserved across primary and metastatic samples and were largely independent of microsatellite instability (MSI) status, underscoring their robustness and biological relevance.

Importantly, the immune–metabolic layer applied in this study should be interpreted as a transcriptomic classifier derived from a limited set of genes linked to immune and metabolic processes, rather than as a comprehensive representation of tumor immune–metabolism. Although combining CMS and immune–metabolic stratification could in principle provide a more refined classification, most survival comparisons within these subgroups did not reach statistical significance, likely due to the reduced sample size after stratification. For this reason, functional interpretation was primarily conducted at the level of immune–metabolic states, which provided sufficient power to identify robust biological differences.

Accordingly, the immune–metabolic states described in this study should be interpreted as coordinated transcriptomic programs rather than as experimentally validated functional states. Although these programs provide a useful framework to generate biological hypotheses regarding metabolic dependencies, stromal interactions, immune-related features and metastatic adaptation, they do not directly demonstrate specific cellular mechanisms, immune cell states or metabolic fluxes. Validation using independent biological datasets, single-cell or spatial profiling, protein-level analyses, metabolomics, isotope tracing and functional perturbation studies will be required to confirm the mechanistic relevance of the IMC-associated programs.

A key finding of this work is the functional heterogeneity observed within CMSs, particularly within CMS1 and CMS2. In CMS2 tumors, immune–metabolic stratification identified a subgroup of patients corresponding to the IMC2 state with significantly worse outcome compared with those associated with IMC3 in limited disease. These observations may reflect distinct metabolic states associated with tumor progression.

In contrast, established macrometastases exhibit increased energetic demands and rely on lactate, lipid and glutamine utilization to sustain oxidative phosphorylation and antioxidant capacity through the pentose phosphate pathway [30,31,32,33,34]. In line with this, the reduced prevalence of IMC2-associated tumors in metastatic cohorts, together with their association with increased chromosomal instability [35], may suggest a shift toward more proliferative metabolic states during disease progression.

In our analysis, the IMC2 state was characterized by transcriptional enrichment of glutamine-related, peroxisomal, lipid metabolic, autophagy and lysosomal programs. These features may reflect an adaptive tumor state enabling survival under nutrient limitation or other microenvironmental stresses, potentially contributing to persistence under therapeutic pressure. In this context, IMC2-associated programs could be relevant for future studies exploring metabolic vulnerabilities related to glutamine utilization, lipid/peroxisomal metabolism, autophagy or lysosomal function. However, these potential functional and therapeutic implications remain hypothesis-generating, as the present study does not include metabolic flux analyses, metabolomics, drug-response assays or experimental perturbation of these pathways.

Consistent with this interpretation, the small subset of tumors retaining immune–metabolic features associated with the IMC2 state in metastatic samples displayed transcriptional patterns reminiscent of dormant or chemotherapy-resistant cancer cell states, characterized by reliance on peroxisomal, lysosomal and lipid metabolic pathways, as well as glutamine-dependent reductive carboxylation [36,37,38]. While the metastatic cohorts analyzed here were enriched for surgically resectable liver and lung metastases, these findings are consistent with previous CMS-based studies limited to selected metastatic populations [12,14,15,16,39,40,41]. However, the apparent conservation of immune–metabolic distributions between primary and metastatic samples should be interpreted with caution, as these cohorts may not capture the full diversity of metastatic colorectal cancer. Future studies including metastases from other anatomical sites, unresectable disease, and heavily pretreated patients will be required to determine whether IMMETCOLS-defined states are conserved across broader metastatic contexts.

Importantly, the characterization of tumor-microenvironment-related features in this study is based on bulk transcriptomic data and reflects gene expression programs associated with stromal and immune components rather than direct measurements of cellular composition. Therefore, these findings should be interpreted as indicative of transcriptomic patterns related to tumor–microenvironment interactions rather than as evidence of the presence or abundance of specific cell populations.

The IMC1 transcriptional program, characterized by glycolytic, hypoxia-related, stromal and immune checkpoint-associated features, is consistent with the broader concept that metabolic reprogramming in colorectal cancer can shape the tumor immune microenvironment through nutrient competition, accumulation of immunosuppressive metabolites and altered immune cell function. A recent review of the metabolism–immune axis in CRC [42] has highlighted how glycolysis, lactate accumulation, lipid and amino acid metabolism, and microbiota-derived metabolites may contribute to immune evasion and therapy resistance. In this context, the increased expression of immune checkpoint-related genes observed in IMC1 tumors may reflect a metabolically and stromally remodeled microenvironment, although our bulk transcriptomic data do not allow us to infer T cell exclusion or functional exhaustion directly.

This observation is relevant in the context of CRC immunotherapy, where immune checkpoint inhibitors have shown clinical benefit mainly in dMMR/MSI-H metastatic CRC, whereas most pMMR/MSS tumors remain poorly responsive [43]. However, our data do not allow us to determine whether IMC1-associated immune checkpoint-related transcriptional programs predict response to immunotherapy.

These findings are consistent with the broader concept that metabolic reprogramming of the CRC tumor immune microenvironment may contribute to immune escape and influence response to immunotherapy. This is particularly relevant given that combinations of immune checkpoint inhibitors with strategies targeting aerobic glycolysis or other metabolic pathways are being actively investigated [22]. Nevertheless, the therapeutic relevance of IMC-associated states remains exploratory and requires validation in therapeutically annotated cohorts and functional experimental models.

Immune–metabolic profiling also provided a refined view of tumor-microenvironment-related transcriptional programs associated with CMSs. CMS4 tumors were predominantly associated with glycolysis, stromal presence, TGFβ signaling and immune-exhausted markers [44].

In contrast, CMS2 and CMS3 tumors, which were enriched in alternative immune–metabolic states, displayed transcriptional profiles consistent with higher oxidative phosphorylation activity and increased metabolic flexibility, together with reduced stromal-associated signatures. These observations are in agreement with previous studies linking chromosomal instability to immune evasion and resistance to immune checkpoint blockade [45,46,47], although such interpretations should be considered at the level of transcriptional programs rather than direct measurements of immune cell composition.

The observed differences in CNA burden across IMMETCOLS clusters suggest that immune–metabolic states may partially reflect differences in chromosomal instability. This is particularly relevant in the context of CMS classification, as CMS2 tumors are typically associated with epithelial, proliferative and CIN-high features, whereas CMS4 tumors are characterized by strong stromal, mesenchymal and TGFβ-related programs. In our TCGA analysis, CMS2 tumors were enriched for the higher-CNA IMC2 and IMC3 states, while CMS4 tumors were enriched for the lower-CNA IMC1 state. Integrating IMMETCOLS with CNA burden may therefore help refine the interpretation of CMS-associated heterogeneity by distinguishing CIN-associated tumor-intrinsic programs from stromal and immune microenvironment-related transcriptional states. However, this analysis remains exploratory and should be validated in larger cohorts with matched transcriptomic and genomic data.

Although CMSs show preferential enrichment for specific immune–metabolic states, the present analyses were not designed for formal comparisons between CMS groups. Therefore, conclusions regarding CMS-specific biological differences should be interpreted with caution.

Notably, CMS1 tumors segregated into distinct immune–metabolic states in comparable proportions. This observation is particularly relevant given that CMS1 tumors are enriched in BRAF-mutant and microsatellite instability-high (MSI-H) colorectal cancers, which are often treated as relatively homogeneous clinical entities. Our findings suggest that immune–metabolic heterogeneity within CMS1 may contribute to variability in tumor-microenvironment-related transcriptional programs and potentially to differences in clinical behavior, thus providing an additional layer of functional stratification beyond genomic classification. The segregation of CMS1 tumors into distinct immune–metabolic states likely reflects the combined contribution of tumor-intrinsic and microenvironment-related transcriptional programs. However, given the use of bulk transcriptomic data, these findings should be interpreted as integrated gene expression patterns rather than as direct evidence of specific cellular or mechanistic drivers.

Finally, the distinct metabolic programs associated with different immune–metabolic states suggest that metabolic dependencies are highly context-dependent and closely linked to tumor-associated transcriptional programs. While the present study is not designed to evaluate therapeutic interventions or predict treatment response, these findings provide a biological rationale for future studies exploring immune–metabolic vulnerabilities within CMS-informed frameworks. Specifically, the current datasets do not allow us to determine whether particular IMC states are more responsive to immunotherapy or metabolic inhibitors. This rationale is supported by a recent review highlighting that CRC metabolic dependencies, including glycolysis, oxidative phosphorylation and mitochondrial bioenergetics, may be therapeutically targeted using metabolic inhibitors or repurposed drugs acting on pathways such as AMPK/mTOR [48]. Nevertheless, the transcriptional features associated with each IMC state may help generate testable hypotheses: IMC1 tumors, characterized by stromal, TGFβ, hypoxia and immune checkpoint-exhausted programs, may be relevant for future studies of TGFβ-targeted therapy INCA33890, a bispecific anti-TGFβ and -PD1; IMC2 tumors, enriched for glutamine-related, peroxisomal, lipid metabolic, autophagy and lysosomal programs, may inform studies with lipid kinase phosphatidylinositol-3-phosphate 5-kinase (PIKfyve) inhibitor (ESK981) [49]; and IMC3 tumors, associated with oxidative phosphorylation and antioxidant programs, may support investigation of mitochondrial inhibitors (IACS-010759) [50] or polo-like kinase 1 inhibitors such as onvansertib [51]. These potential therapeutic implications remain exploratory and require validation in prospective cohorts with treatment-response data and functional experimental models.

Overall, immune–metabolic profiling complements the CMS classification by revealing functional heterogeneity that is not captured by transcriptomic subtype classification alone. By integrating this additional layer of information, our results contribute to improving the biological interpretability of colorectal cancer subtypes and highlight the importance of considering metabolic context when studying tumor heterogeneity.

## 5. Conclusions

In conclusion, integrating IMMETCOLS-based stratification into the established Consensus Molecular Subtype (CMS) framework improves the interpretation of colorectal cancer heterogeneity. This classification reveals biologically meaningful differences within CMSs, including variation in transcriptomic programs related to metabolism, stromal features and clinical behavior that are not captured by CMS classification alone.

These findings support the value of IMMETCOLS as a complementary transcriptomic classifier within existing molecular classification frameworks. For example, these hypotheses could be explored in future studies evaluating immune/stroma-targeted strategies, inhibitors of autophagy–lysosomal or lipid metabolic adaptation, mitochondrial metabolism inhibitors, or cell-cycle/metabolic modulators in therapeutically annotated cohorts and experimental models.

## Data Availability

All data generated or analyzed during this study are included in this published article and its Supplementary Information files. Results and scripts are publicly available at https://github.com/smadurga/CRC_immune_metabolic_profiling (accessed on 4 April 2026).

## Supplementary Materials

The following supporting information can be downloaded at https://www.mdpi.com/article/10.3390/biology15141128/s1, Figure S1A: Heatmap of patients classified according to IMC group for the GSE1 and TCGA sets (A) GSE1 (*n* = 1328). (B) TCGA (*n* = 456). Gene expression values are range-scaled between −3 and +3; Figure S1B: Heatmap of the IMMETCOLS gene signature used to classify patients of the GSE2 set into each cluster. (A) MCC – Primary Tumor (*n* = 333). (B) MCC – Metastasis (*n* = 184). (C) Consortium – Primary Tumor *n* = 545). (D) Consortium – Metastasis (*n* = 72). Gene expression values are range-scaled between −3 and +3. Figure S2. Expanded heatmap of selected metabolic and tumor-microenvironment-related genes across IMMETCOLS clusters in the GSE1, TCGA, MCC PRIM, MCC MET, Consort PRIM and Consort MET cohorts. Table S1: Clinical and histopathological characteristics of patients of the GSE1 and TCGA sets; Table S2: Clinical and histopathological characteristics of patients of the GSE2 set (GSE131418); Table S3: Multivariate Cox proportional hazards model for overall survival in CMS2 stage I–III patients from the GSE1 cohort; Table S4: Excel file with statistical parameters of manuscript figures.

## Abbreviations

The following abbreviations are used in this manuscript:

BCKAs	Branched-chain keto acids
CAF	Cancer-associated fibroblast
CMS	Consensus Molecular Subtype
CNA	Copy number alteration
CRC	Colorectal cancer
EMT	Epithelial–mesenchymal transition
FDR-BH	False discovery rate—Benjamini–Hochberg
GDC	Genome Data Commons
GEO	Gene Expression Omnibus
GLUL	Glutamine synthetase
MET	Metastatic
MSI	Microsatellite instability
MSI-H	Microsatellite instability-high
MSI-L	Microsatellite instability-low
MSS	Microsatellite stability
OS	Overall survival
OXPHOS	Oxidative phosphorylation
PLK1	Polo-like kinase 1
PPP	Pentose phosphate pathway
PRIM	Primary
