# Supplementary material for: Immune–Metabolic Profiling Reveals Functional Heterogeneity Within Colorectal Cancer Consensus Molecular Subtypes
Source: Biology (Basel). 2026 Jul 10;15(14):1128. doi: 10.3390/biology15141128 (PMC13403527; doi:10.3390/biology15141128)

SUPPLEMENTARY MATERIAL

Immune–Metabolic Profiling Reveals Functional Heterogeneity Within Colorectal Cancer Consensus Molecular Subtypes

Sergio Madurga [1,2], David López-Blanco [2,3,4], Carles Foguet [5,6], Sara Lahoz [2,7], Helena Oliveres [8], Reinaldo Moreno [8], Teresa Gorria [8], Leire Pedrosa [8], Silvia Marin [2,3,4], Mariam Rojas [8], Jordi Camps [2,7,9], Francesc Mas [1], Joan Maurel [2,7,8,*] and Marta Cascante [2,3,4,*]

1 Department of Material Science and Physical Chemistry and Research Institute of Theoretical and Computational Chemistry of University of Barcelona (IQTCUB), Universitat de Barcelona, 08028 Barcelona, Spain; s.madurga@ub.edu (S.M.); fmas@ub.edu (F.M.)
2 Centro de Investigación Biomédica en Red de Enfermedades Hepáticas y Digestivas (CIBEREHD), Instituto de Salud Carlos III (ISCIII), 28029 Madrid, Spain; dlopezbl@ub.edu (D.L.-B.); salahoz@recerca.clinic.cat (S.L.); silviamarin@ub.edu (S.M.); jcamps@clinic.cat (J.C.)
3 Department of Biochemistry and Molecular Biomedicine, Faculty of Biology, Universitat de Barcelona, 08028 Barcelona, Spain
4 Institute of Biomedicine of Universitat de Barcelona (IBUB), Universitat de Barcelona, 08028 Barcelona, Spain
5 British Heart Foundation Cardiovascular Epidemiology Unit, Department of Public Health and Primary Care, University of Cambridge, Cambridge CB2 0SR, UK; df545@cam.ac.uk
6 Victor Phillip Dahdaleh Heart and Lung Research Institute, University of Cambridge, Cambridge CB2 0SR, UK
7 Gastrointestinal and Pancreatic Oncology Group, Institut D'Investigacions Biomèdiques August Pi i Sunyer (IDIBAPS), 08036 Barcelona, Spain
8 Translational Genomics and Targeted Therapeutics in Solid Tumors Group, IDIBAPS, Medical Oncology Department, Hospital Clínic of Barcelona, Universitat de Barcelona, 08036 Barcelona, Spain; helena.oliveres@uza.be (H.O.); rzambrano@clinic.cat (R.M.); tgorria@clinic.cat (T.G.); leire.pedrosa@iibb.csic.es (L.P.); rojasp@recerca.clinic.cat (M.R.)
9 Faculty of Medicine, Autonomous University of Barcelona, 08193 Barcelona, Spain
* Correspondence: jmaurel@clinic.cat (J.M.); martacascante@ub.edu (M.C.)

Table S1: Clinical and histopathological characteristics of patients of the GSE1 and TCGA sets

GSE1

**Characteristics**	**CMS1**	**CMS2**	**CMS3**	**CMS4**	**NA**
**Number**	251	462	147	187	281
**Mean age (SD)**	68 (15)	67 (13)	68 (11)	62 (13)	65 (14)
**Sex (male/female)**	90/135	243/179	73/59	92/81	139/115
**IMC cluster (%)**					
IMC1	117 (47)	43 (9)	4 (3)	168 (90)	104 (37)
IMC2	16 (6)	93 (20)	40 (27)	7 (4)	30 (11)
IMC3	118 (47)	326 (71)	103 (70)	12 (6)	147 (52)
**Location (%)**					
Left side	35 (22)	229 (75)	40 (40)	76 (58)	130 (69)
Right side	123 (78)	76 (25)	58 (59)	56 (42)	58 (31)
**dMMR (%)**	68 (75)	4 (2)	3 (4)	2 (2)	9 (7)
RAS** (%)	28 (28)	55 (26)	54 (82)	34 (37)	67 (51)
BRAF* (%)	43 (44)	0 (0)	2 (3)	6 (7)	6 (5)

TCGA

**Characteristics**	**CMS1**	**CMS2**	**CMS3**	**CMS4**	**NA**
**Number**	64	234	39	41	78
**Mean age (SD)**	72 (13)	67 (12)	64 (13)	61 (12)	68 (14)
**Sex (male/female)**	23/41	130/102	23/16	21/20	43/35
**IMC cluster (%)**					
IMC1	31 (48)	32 (14)	3 (8)	41 (100)	32 (41)
IMC2	7 (11)	48 (21)	9 (23)	0 (0)	4 (5)
IMC3	26 (41)	154 (66)	27 (69)	0 (0)	42 (54)
**Location (%)**					
Left side	8 (13)	105 (45)	8 (21)	11 (27)	25 (32)
Right side	44 (69)	69 (29)	24 (62)	17 (41)	35 (45)
**dMMR (%)**	57 (89)	1 (0.4)	6 (15)	4 (10)	12 (15)
***RAS** (%)**	11 (21)	79 (35)	26 (76)	15 (38)	37 (52)
BRAF** (%)	37 (70)	3 (2)	3 (9)	5 (13)	5 (7)

NA: not assigned

Table S2: Clinical and histopathological characteristics of patients of the GSE2 set (GSE131418).

**Characteristics**	**CMS1**	**CMS2**	**CMS3**	**CMS4**	**NA**
**Number**	136	227	102	343	276
**Mean age (SD)**	71 (13)	64 (13)	67 (14)	63 (13)	63 (13)
**Sex (male/female)**	40/96	164/113	53/49	192/151	151/125
**IMC cluster (%)**					
IMC1	39 (29)	7 (3)	2 (2)	279 (81)	61 (22)
IMC2	7 (5)	36 (13)	20 (20)	26 (8)	34 (12)
IMC3	90 (66)	234 (84)	80 (78)	38 (11)	181 (66)
**Location (%)**					
Left side	26 (19)	203 (74)	48 (48)	214 (64)	152 (57)
Right side	108 (81)	70 (26)	52 (52)	123 (36)	113 (43)
**Primary/metastasis**	128/8	211/66	94/8	254/89	191/85
Pre-treatment	123	216	82	155	173
Post-treatment	13	61	20	188	103
**Metastatic site (liver/lung)**	7/1	52/14	7/1	73/16	57/28

NA: not assigned

Table S3. Multivariate Cox proportional hazards model for overall survival in CMS2 stage I–III patients from the GSE1 cohort.

Number of observations: 286
Number of events observed: 73
Concordance: 0.68
Partial AIC: 717.67
log-likelihood ratio test: 31.33 on 6 df
-log2(p) of ll-ratio test: 15.48

**Characteristics**	**HR**	**HR lower 95%**	**HR upper 95%**	**z**	**p**
**age**	1.04	1.02	1.06	3.47	<0.005
IMC1	0.43	0.16	1.15	-1.69	0.09
IMC3	0.50	0.30	0.83	-2.66	0.01
Stage I	0.18	0.04	0.75	-2.36	0.02
Stage II	0.85	0.53	1.37	-0.66	0.51
Left side	0.82	0.48	1.38	-0.76	0.45

IMC2 was used as the reference category. The model was adjusted for age, tumor stage and tumor sidedness. The reduced number of observations reflects availability of complete clinicopathological data for all covariates included in the model.

Figure S1A. Heatmap of patients classified according to IMC group for the GSE1 andTCGA sets. (A) GSE1 (n = 1328). (B) TCGA (n = 456). Gene expression values are range-scaled between −3 and +3.

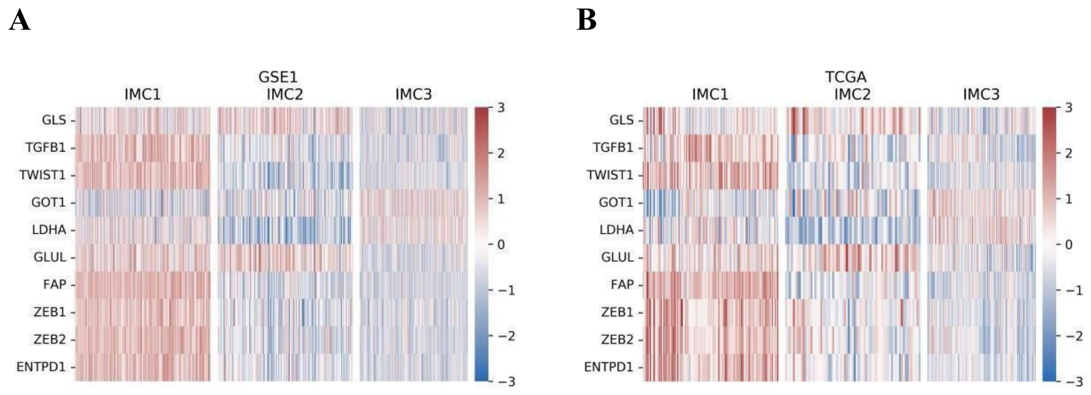

Figure S1B. Heatmap of the IMMETCOLS gene signature used to classify patients of theGSE2 set into each cluster. (A) MCC – Primary Tumor (n = 333). (B) MCC – Metastasis (n = 184). (C) Consortium – Primary Tumor n = 545). (D) Consortium – Metastasis (n = 72). Gene expression values are range-scaled between -3 and +3.

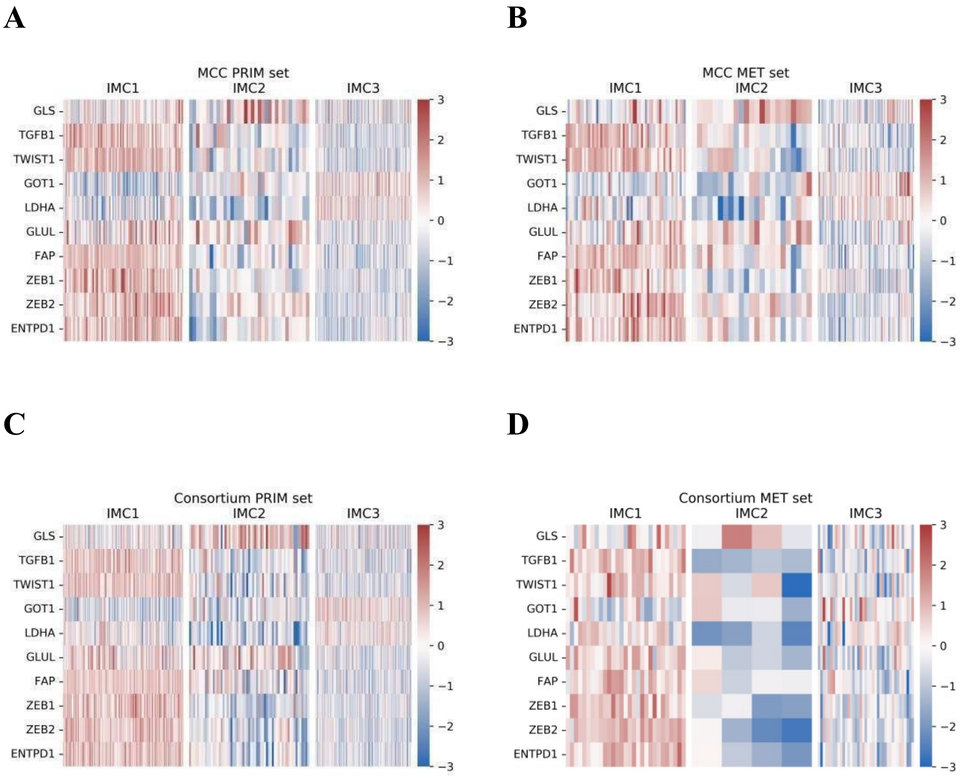

Figure S2A. Expanded heatmap of selected metabolic and tumor-microenvironment-related genes across IMMETCOLS clusters in the GSE1 cohort.
Average scaled gene expression is shown for IMC1, IMC2 and IMC3 tumors in the GSE1 cohort. Genes are grouped into the main programs discussed in the manuscript: glycolysis/lactate transport and hypoxia-related metabolism; amino acid transport, regulatory and hexosamine/glycosylation-related metabolism; stromal and collagen-associated programs; glutamine/peroxisomal/autophagy–lysosomal and transmembrane-associated programs; and glucose uptake/glycolysis, mitochondrial/OXPHOS, one-carbon, PPP and cell-cycle-related programs. Red denotes higher relative expression and blue denotes lower relative expression after gene-wise scaling.

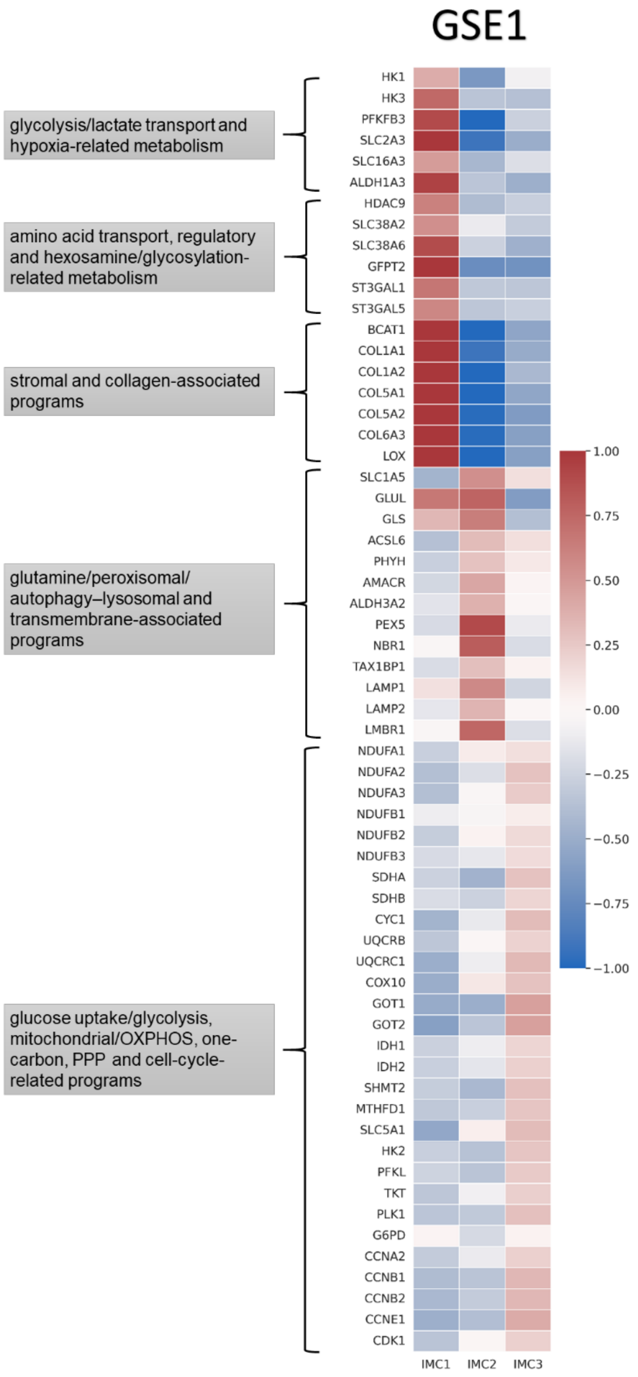

Figure S2B. Expanded heatmap of selected metabolic and tumor-microenvironment-related genes across IMMETCOLS clusters in the TCGA cohort.
Average scaled gene expression is shown for IMC1, IMC2 and IMC3 tumors in the TCGA cohort. Genes are grouped into the main programs discussed in the manuscript: glycolysis/lactate transport and hypoxia-related metabolism; amino acid transport, regulatory and hexosamine/glycosylation-related metabolism; stromal and collagen-associated programs; glutamine/peroxisomal/autophagy–lysosomal and transmembrane-associated programs; and glucose uptake/glycolysis, mitochondrial/OXPHOS, one-carbon, PPP and cell-cycle-related programs. Red denotes higher relative expression and blue denotes lower relative expression after gene-wise scaling.

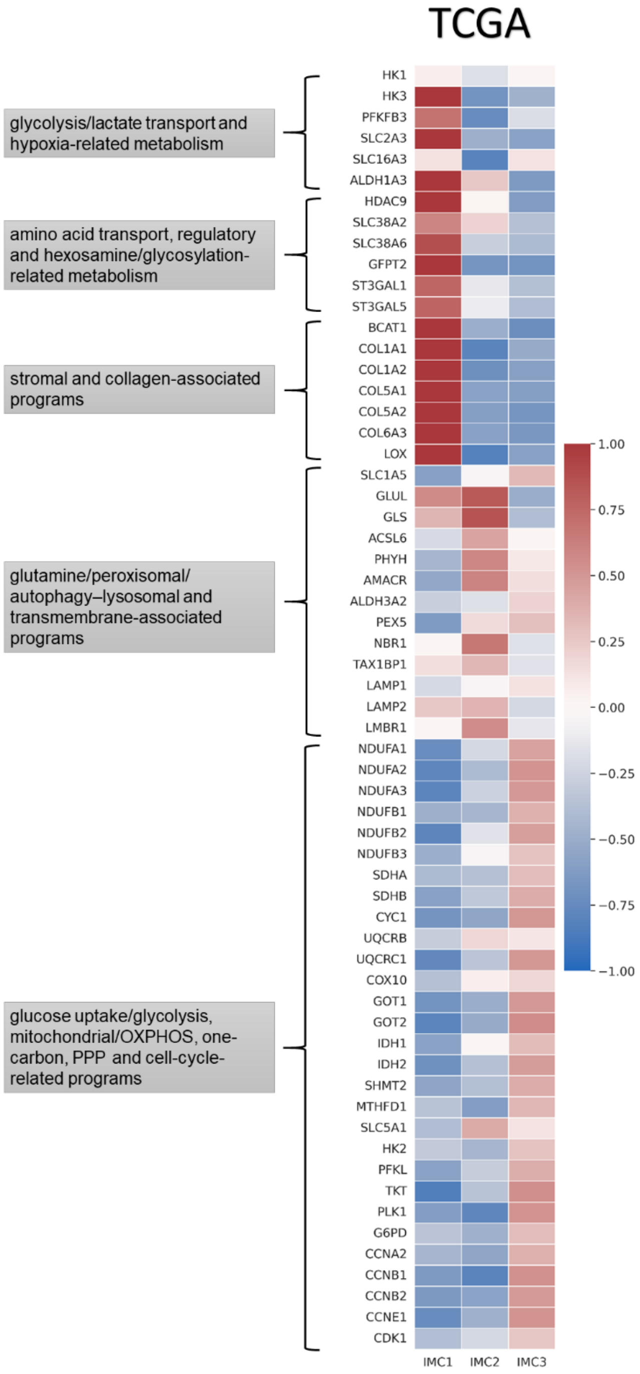

Figure S2C. Expanded heatmap of selected metabolic and tumor-microenvironment-related genes across IMMETCOLS clusters in the MCC PRIM cohort.
Average scaled gene expression is shown for IMC1, IMC2 and IMC3 tumors in the MCC PRIM cohort. Genes are grouped into the main programs discussed in the manuscript: glycolysis/lactate transport and hypoxia-related metabolism; amino acid transport, regulatory and hexosamine/glycosylation-related metabolism; stromal and collagen-associated programs; glutamine/peroxisomal/autophagy–lysosomal and transmembrane-associated programs; and glucose uptake/glycolysis, mitochondrial/OXPHOS, one-carbon, PPP and cell-cycle-related programs. Red denotes higher relative expression and blue denotes lower relative expression after gene-wise scaling.

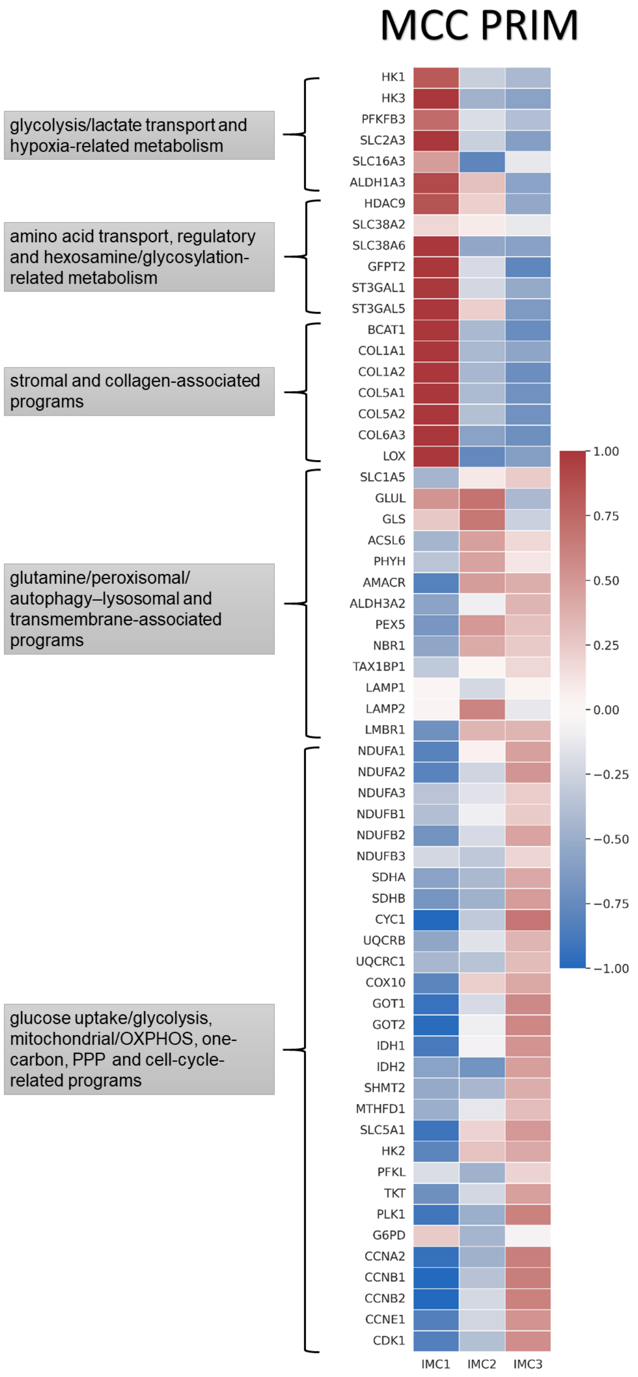

Figure S2D. Expanded heatmap of selected metabolic and tumor-microenvironment-related genes across IMMETCOLS clusters in the MCC MET cohort.
Average scaled gene expression is shown for IMC1, IMC2 and IMC3 tumors in the MCC MET cohort. Genes are grouped into the main programs discussed in the manuscript: glycolysis/lactate transport and hypoxia-related metabolism; amino acid transport, regulatory and hexosamine/glycosylation-related metabolism; stromal and collagen-associated programs; glutamine/peroxisomal/autophagy–lysosomal and transmembrane-associated programs; and glucose uptake/glycolysis, mitochondrial/OXPHOS, one-carbon, PPP and cell-cycle-related programs. Red denotes higher relative expression and blue denotes lower relative expression after gene-wise scaling.

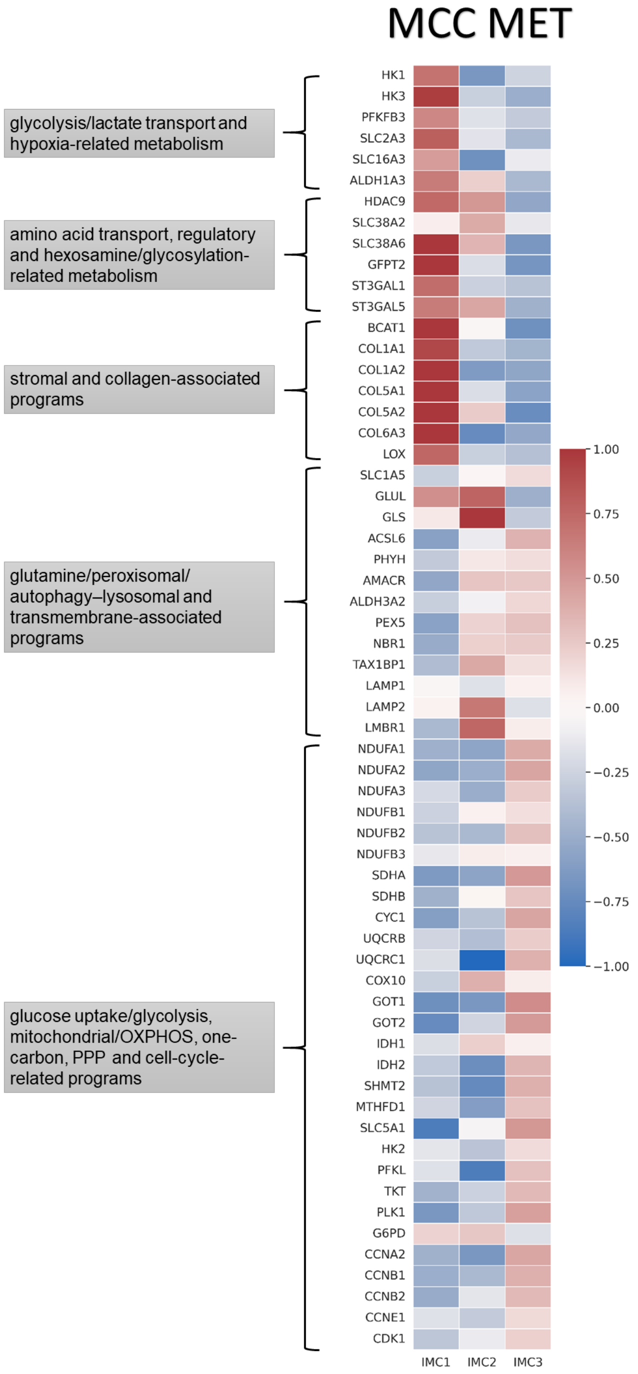

Figure S2E. Expanded heatmap of selected metabolic and tumor-microenvironment-related genes across IMMETCOLS clusters in the Consort PRIM cohort.

Average scaled gene expression is shown for IMC1, IMC2 and IMC3 tumors in the Consort PRIM cohort. Genes are grouped into the main programs discussed in the manuscript: glycolysis/lactate transport and hypoxia-related metabolism; amino acid transport, regulatory and hexosamine/glycosylation-related metabolism; stromal and collagen-associated programs; glutamine/peroxisomal/autophagy–lysosomal and transmembrane-associated programs; and glucose uptake/glycolysis, mitochondrial/OXPHOS, one-carbon, PPP and cell-cycle-related programs. Red denotes higher relative expression and blue denotes lower relative expression after gene-wise scaling.

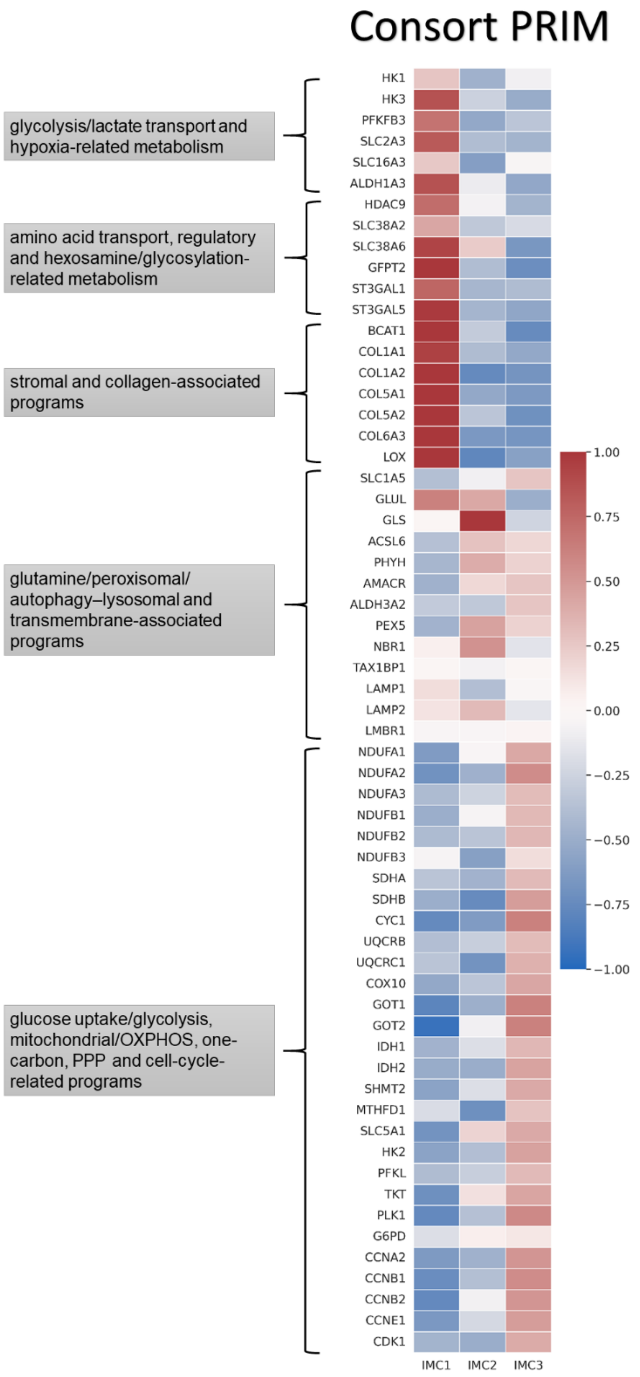

Figure S2F. Expanded heatmap of selected metabolic and tumor-microenvironment-related genes across IMMETCOLS clusters in the Consort MET cohort.

Average scaled gene expression is shown for IMC1, IMC2 and IMC3 tumors in the Consort MET cohort. Genes are grouped into the main programs discussed in the manuscript: glycolysis/lactate transport and hypoxia-related metabolism; amino acid transport, regulatory and hexosamine/glycosylation-related metabolism; stromal and collagen-associated programs; glutamine/peroxisomal/autophagy–lysosomal and transmembrane-associated programs; and glucose uptake/glycolysis, mitochondrial/OXPHOS, one-carbon, PPP and cell-cycle-related programs. Red denotes higher relative expression and blue denotes lower relative expression after gene-wise scaling.

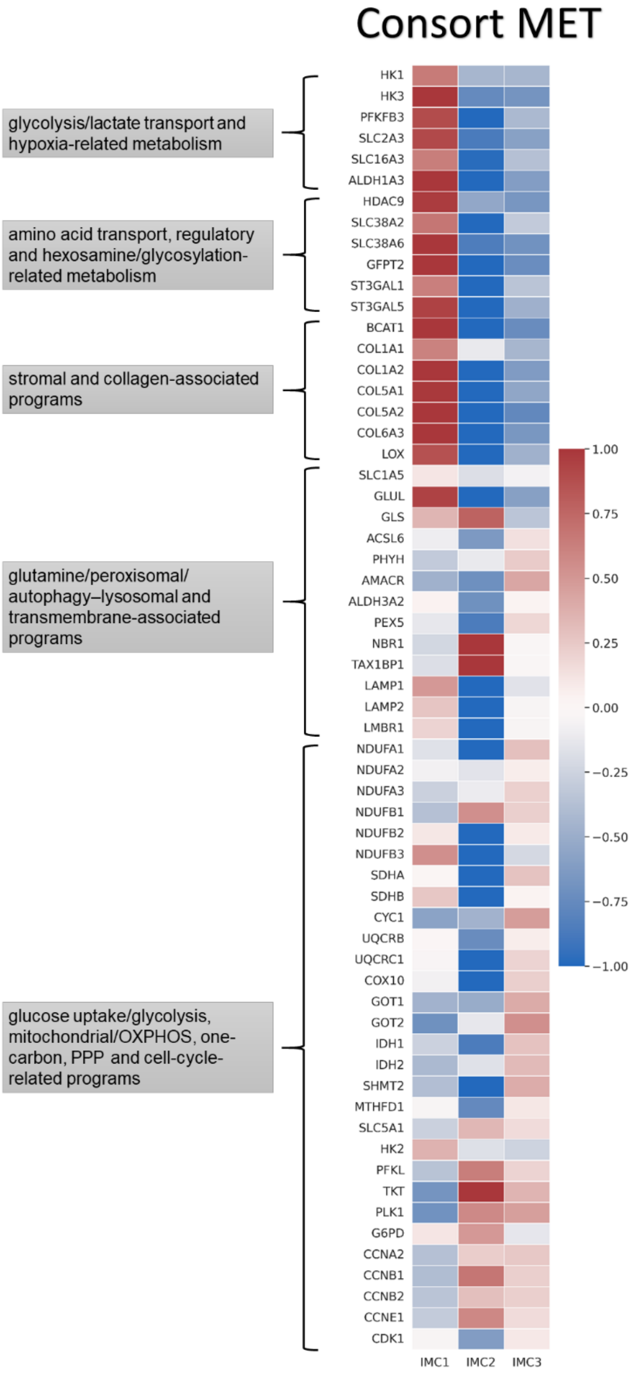

Supplement: Supplementary file 1 [file biology-15-01128-s001.zip › biology-4378828-supplementary.pdf]
